# A Novel Asp121Asn Mutation of Myelin Protein Zero Is Associated with Late-Onset Axonal Charcot-Marie-Tooth Disease, Hearing Loss and Pupil Abnormalities

**DOI:** 10.3389/fnagi.2016.00222

**Published:** 2016-09-22

**Authors:** Xiaohui Duan, Weihong Gu, Ying Hao, Renbin Wang, Hong Wen, Shaojie Sun, Jinsong Jiao, Dongsheng Fan

**Affiliations:** ^1^Department of Neurology, Peking University Third HospitalBeijing, China; ^2^Department of Neurology, China-Japan Friendship HospitalBeijing, China; ^3^Department of Radio-Diagnosis, China-Japan Friendship Hospital, BeijingChina

**Keywords:** Charcot–Marie–Tooth disease, hearing loss, myelin protein zero, novel mutation, pupil abnormalities

## Abstract

Myelin protein zero (MPZ) is a major component of compact myelin in peripheral nerves. Mutations in MPZ have been associated with different Charcot–Marie–Tooth disease (CMT) phenotypes (CMT1B, CMT2I/J, CMTDI), Dejerine–Sottas syndrome, and congenital hypomyelination neuropathy. Here, we report phenotypic variability in a four-generation Chinese family with the MPZ mutation Asp121Asn. Genetic testing was performed on nine family members and 200 controls. Clinical, electrophysiological and skeletal muscle MRI assessments were available for review in six family members. A novel heterozygous missense mutation, Asp121Asn, was observed in five affected members of the family. Unaffected relatives and 200 normal controls were without the mutation. Four of the affected members of the family displayed late-onset, predominantly axonal sensory and motor neuropathy, pupil abnormalities, and progressive sensorineural hearing loss. One young affected member presented with Argyll–Robertson pupils and diminished deep tendon reflexes in the lower limbs. The MPZ mutation Asp121Asn may be associated with late-onset axonal neuropathy, early onset hearing loss and pupil abnormalities. Our report expands the number and phenotypic spectrum of MPZ mutations.

## Introduction

Charcot–Marie–Tooth disease (CMT) is a genetically heterogeneous group of disorders affecting the peripheral nervous system. The number of disease genes identified in CMT has expanded rapidly over the past few decades (Rossor et al., 2013). Mutations in the myelin protein zero (*MPZ*) gene are one of the most frequent causes of CMT. To date, 190 different mutations have been documented^1^. In this report, we present five patients from a four-generation Chinese family who contain a novel missense mutation in *MPZ*.

## Patients and Methods

### Patients

**FIGURE 1:**
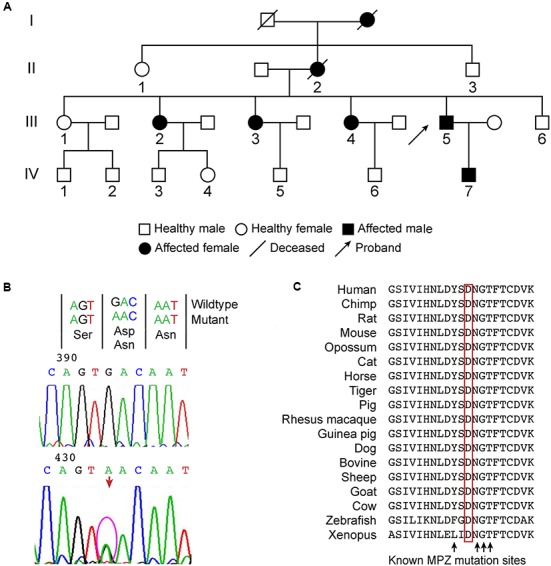
**(A)** Family tree of a Chinese family of Asian descent affected by peripheral neuropathy, pupil abnormalities, and progressive sensorineural hearing loss. The family included 7 affected and 10 unaffected members over four generations. The proband is member III-5. The other family members, II-1, II-3 III-1-4, III-6, and IV-7, were examined clinically or electrophysiologically and received genetic testing. **(B)** A novel heterozygous missense mutation c.361G>A was identified in *MPZ* exon 3, which caused the Asp121Asn substitution in the extracellular domain. **(C)** The position of the mutation is highly conserved in vertebrates, juxtaposed with known mutations, and located within the critical disulfide bonding domain responsible for the adhesive properties of MPZ.

The family included 7 affected and 10 unaffected members over four generations (**Figure 1A**). This study was approved by the ethical committees of the China-Japan Friendship Hospital and the Peking University Third Hospital. Written informed consent was obtained from all participants.

The proband (III-5), a 45-year-old man, presented with a 9-year history of progressive difficulty in walking coupled with hearing loss. At 36 years of age, he developed numbness and weakness in his legs. He also noticed a progressive bilateral hearing loss with tinnitus. Hand-grip weakness and numbness became evident 3 years after onset. The disease progressed over the next few years, resulting in marked wasting of the calf muscles and foot drop. He required hearing aids at the age of 45. Neurological examination indicated muscle weakness and atrophy in the distal legs and hands. The muscle strength of the anterior tibial and gastrocnemius muscles was 3 of 5 (Medical Research Council [MRC] scale). The muscle strength of the foot and ankle flexor–extensor muscles was 0 of 5, and pes cavus and steppage gait were present. A score of 4 of 5 was recorded for the finger abductor and adductor muscles. Deep tendon reflexes were absent in the lower limbs and diminished in the upper limbs. Pyramidal tract signs were not observed. There was mild reduction of perception to touch, pain and temperature in glove-stock distribution and moderate reduction of vibration sense in the feet. The pupils were asymmetric (left, 4 mm; right, 2 mm) with a slow response for the light reflex and accommodation reaction.

Patient (IV-7), the proband’s 24-year-old son, did not complain of paresthesia or weakness of his limbs. He only noticed that he was slightly unsteady when walking on his heels and toes. He denied hearing and vision problems. On examination, positive signs included pupil abnormalities (pupils had a symmetrical but irregular size of 2 mm and were unresponsive to light but exhibited normal constriction on convergence) and diminished deep tendon reflexes in the lower limbs. The muscle strength of the limbs was 5 of 5 on the MRC scale. Sensory examinations were unremarkable.

Patient (II-2), the proband’s mother, died at the age of 66. She suffered from hearing loss at age 38 and then developed gait difficulties with decreased sensation in both of her lower limbs at the age of 40. She became completely deaf at the age of 50. She also had a history of recurrent coughing spasms. These episodes would typically last for 20–30 min and end in vomiting or syncope. The deceased mother also displayed gait disturbances and hearing impairment.

Patients (III-2, III-3, III-4) were affected between the ages of 36 and 40 with symptoms comparable to those of the proband (**Table 1**). All of the children of these patients are currently asymptomatic.

**Table 1:** Clinical features in individuals with genetically confirmed neuropathy.

Pedigree	Sex	Onset age (years)	Clinical features	Muscle strength		Reflexes	Hearing loss age (years)
				UL	LL
II-2	F	40	Gait difficulties, hypoesthesia of distal lower limbs, cough spasms	NA	NA	NA	38
III-2	F	38	Weakness and numbness of distal lower limbs, pes cavus, pupil abnormalities	4	0–3	Absent in ankle and knee	37
III-3	F	37	Distal hypoesthesia and paresthesia, distal weakness, pes cavus, Adie’s pupil	4	0–2	Diminished in the upper limbs and absent in the lower limbs	38
III-4	F	36	Bilateral foot drop and distal dysesthesia, pes cavus, pupil abnormalities	5	0–2	Absent in ankle and knee	36
III-5	M	36	Distal weakness and paresthesia, pes cavus, Adie’s pupil	4	0–3	Diminished in the upper limbs and absent in the lower limbs	36
IV-7	M	N	Argyll–Robertson pupils	5	5	Diminished in the lower limbs	N

*F, female; M, male; UL, upper limbs; LL, lower limbs; N, no finding; NA, not available for clinical studies.*

### Genetic Analysis

Duplication of the fragment that contains *PMP22* was performed with multiplex ligation-dependent probe amplification (SALSA MLPA kit P033, MRC-Holland). The coding and splicing of the site-flanking regions of *PMP22*, *MFN2*, *GJB1*, and *MPZ* were PCR amplified with intronic primers and directly sequenced using Applera BigDye version 3.1 (Applied Biosystems, Foster City, CA, USA) and the automated sequencer ABI 3730XL (Applied Biosystems). The amplicon sequences were aligned by SeqMan Pro to the published human gene sequences in the NCBI database^2^ with 7.1.0 (DNASTAR Inc., Madison, WI, USA). All nucleotide differences were compared to dbSNP^3^ and to the human genetic mutation databases IPNMDB^4^ and HGVS^5^. The *in silico* pathogenicity prediction tools Provean^6^, PolyPhen^7^ and Mutation Taster^8^ were used to further validate the mutations.

## Results

### Clinical Findings

All patients presented with pes cavus, distal sensory loss, and gradually progressive weakness of the lower and upper extremities, and they developed bilateral hearing loss. The proband plus patients III-2-4 exhibited pupillary abnormalities. Patient II-2 had a record of coughing spasms, and the autonomic nervous system may have been involved. Patient IV-7 was asymptomatic, and examination only identified diminished deep tendon reflexes in the lower limbs and Argyll–Robertson pupils.

Patients III-2, III-5, and IV-7 participated in electrophysiological tests (**Table 2**). Patient III-2 and proband III-5 displayed a predominantly axonal neuropathy with only mild myelin changes and marked abnormalities in the lower limb motor nerves. Proband III-5 exhibited minor slowing in motor conduction velocity and decreased compound motor action potentials (CMAP) in the median nerve. The amplitudes of sensory nerve action potentials (SNAP) were predominantly reduced with mild slowing in the sensory conduction velocities of the median and ulnar nerves. The lower limb nerves exhibited severely decreased or absent CMAP and SNAP with slowing nerve conduction velocities. Patient III-2 showed results similar to those of the proband. The brainstem auditory evoked potentials (BAEP) of Patients III-2 and III-5 showed evidence of peripheral lesions. Audiometry for Patients III-2 and III-5 indicated symmetric sensorineural hearing loss in the 30–50 dB hearing level (HL) at moderate frequencies and severe hearing loss in the 50–80 dB HL at high frequencies. All electrophysiological tests for Patient IV-7 (at age 24) were preserved.

**Table 2:** Electrophysiological features in three affected members of the family.

	Proband III-5 (45 years)			Patient III-2 (50 years)			Patient IV-7 (24 years)		
Nerve	MCV, m/s	DL, ms	CMAP, mV	MCV, m/s	DL, ms	CMAP, mV	MCV, m/s	DL, ms	CMAP, mV
**Median**									
E-W	46.5↓	8.3	4.21↓	55.1	7.1	14.4	57.8	5.8	15.4
W-APB		4↓	3.71↓		3.2	15.7		2.6	16.9
**Ulnar**									
E-W	53	6.5	6.9	53.8	6.4	8.9	62.5	5.3	13.1
W-ADM		2.67	6.4	2.5		7.1		2.1	13.6
**Tibial**									
K-A	37.3↓	12.4	0.16↓↓	33.9↓	15.6	0.49↓↓	46.4	10.9	4.8
A-FHB		4.5	0.22↓↓		4.9	2.26↓		3.8	7.1
**Peroneal**									
K-A	NR	NR	NR		ND			ND	
A-EDB		NR	NR		ND			ND	

**Nerve**	**SCV, m/s**		**SNAP, μV**	**SCV, m/s**		**SNAP, μV**	**SCV, m/s**		**SNAP, μV**

**Median**									
IIIF-W	35.5↓		2.6↓↓	50		2.9↓↓	59.2		5.8
**Ulnar**									
VF-W	37.1↓		1.6↓↓	40.7		2.4↓↓	54.7		5.3
**Sural**									
A-sural	NR		NR	NR		NR	50.1		4

*A, ankle; ADM, abductor digiti minimi; APB, abductor pollicis brevis; CMAP, compound muscle action potential; DL, distal latency; E, elbow; EDB, extensor digitorum brevis; FHB, flexor hallucis brevis; III F, third finger; K, knee; MCV, motor conduction velocity; SCV, sensory conduction velocity; SNAP, sensory nerve action potential; V F, fifth finger; W, wrist; NR, no response; ND, not performed. ↓↓, remarkable decrease or reduction. ↓, mild decrease or reduction.*

Skeletal muscle MRI of the lower limbs was performed on proband III-5 and his son IV-7 (**Supplemental Figure 1**). Proband III-5 displayed extensive and symmetric fatty atrophy of the four lower leg muscle compartments, particularly in the anterolateral compartments and gastrocnemius muscles. Fatty infiltration was classified as stage IV. Patient IV-7 exhibited subtle and symmetric fatty infiltration of the soleus and gastrocnemius muscles. Fatty infiltration was classified as between stages 0 and I. The MRI images of the proband and his son indicated that fatty infiltration was related to disease duration. These findings are similar to the reported features of axonal CMT2 neuropathy (Gallardo et al., 2009).

### Genetic Analysis

Molecular analysis of *PMP22* ruled out the presence of a duplication or deletion. Direct sequence analysis of *PMP22*, *GJB-1*, *MFN2*, and *MPZ* was performed for proband III-5. A novel heterozygous missense mutation c.361G>A was identified in *MPZ* exon 3 (**Figure 1B**), which caused the Asp121Asn substitution in the extracellular domain. This mutation was subsequently observed in all of the affected patients who were examined but not in the four unaffected relatives (II-1, II-3, III-1, III-6). Direct testing of asymptomatic children IV-3-6 was not authorized by their parents. The nucleotide substitution was absent in 200 control chromosomes from unrelated Chinese individuals.

Provean (deleterious, *p* = -2.99), Mutation Taster (disease-causing probability score = 1) and PolyPhen2 (probably damaging, PSIC: 0.995) all predicted that the mutation was damaging. This amino acid is highly conserved in vertebrates, juxtaposed with known mutations and found within the critical disulfide bonding domain responsible for the adhesive properties of MPZ (**Figure 1C**).

## Discussion

Patients with *MPZ* mutations exhibit a wide range of phenotypic variability beyond the peripheral nervous system, including pupillary abnormalities, hearing loss, diaphragmatic weakness or chronic cough, restless-leg-like symptoms and multiple sclerosis (De Jonghe et al., 1999; Baloh et al., 2004; Kilfoyle et al., 2006). Such variability may be related to dosage of the MPZ protein or the position and nature of the MPZ mutation. In this report, we described a large Chinese family with late-onset axonal neuropathy, pupil abnormalities, and hearing loss associated with a novel mutation in *MPZ*.

In this family, similar symptoms began around the fourth decade of life. The patients’ (II-2, III-2-5) symptoms began with distally accentuated sensory and motor symptoms in limbs, or hearing impairment, followed by the appearance and subsequent development of lesion-associated symptoms. Patient II-2 also had a history of recurrent coughing spasms. Similar cases have been reported that are associated with the Thr124Met mutation in *MPZ* (Baloh et al., 2004). Patient IV-7 was asymptomatic at age 24. Our findings indicate that until adulthood, his disease may remain quiescent with only unnoticeable pupillary abnormalities and early selective length-dependent hyporeflexia. As reported previously, abnormal or abolished pupillary reflex may be the first sign of CMT disease associated with MPZ mutation, as asymptomatic carriers may present pupillary abnormalities before they reach the age of onset (De Jonghe et al., 1999; Misu et al., 2000). Furthermore, a previous case reported that the Thr124Met mutation in *MPZ* results in the early involvement of the autonomic nervous system and the late involvement of the somatic peripheral nervous system (Baloh et al., 2004). Our study suggests that *MPZ* may also be involved in the autonomic system, which includes unmyelinated post-ganglionic sympathetic and parasympathetic axons. The pupils exhibited a slow but preserved miosis associated with accommodative effort, suggesting damage to the post-ganglionic parasympathetic fibers (Murphy et al., 2011). Similar pupillary dysfunction resulting in a lack of response to light but a retained response to accommodation was reported in patients with the Thr124Met, His81Tyr, and Val113Phe MPZ mutations (Bienfait et al., 2002). Because tonic pupils are more often observed in axonal rather than demyelinating forms of CMT (Hattori et al., 2003), MPZ must be critical for the maintenance of axonal function in addition to its role as a structural protein in myelin.

Hearing impairment appears to be an additional feature of late-onset CMT2 disease. The documented MPZ mutations associated with variable degrees of hearing loss are Thr124Met, Asp75Val, Glu97Val, Tyr145Ser, Pro105Thr, and His39Pro (De Jonghe et al., 1999; Misu et al., 2000; Starr et al., 2003; Kilfoyle et al., 2006; Kabzińska et al., 2007). In this study, BAEP and audiometry findings indicated sensorineural deafness in the affected family numbers with a novel Asp121Asn mutation. Based on a recent study investigating the Tyr145Ser mutation in *MPZ*, in sural and auditory/vestibular nerves, the pathological findings indicated a loss of large myelinated fibers and thinning of the myelin sheath of the remaining axons, consistent with a primary axonal disorder with incomplete remyelination (Starr et al., 2003). Price et al. (1993) divided the denervated muscles for the lower legs into “P-type” (peroneal) and “T-type” (tibial) depending on the predominant muscles affected by neuropathy. The family in the present study displayed a characteristic pattern of “partial T-type” muscles abnormalities. The observed normal thigh musculature versus distally accentuated fatty infiltration of the lower leg muscle bellies in proband III-5 also concurs with length-dependent axonopathy.

Most MPZ mutations are located within the extracellular or transmembrane domain of the mature MPZ protein. In this report, the missense mutation identified in exon 3 of *MPZ* results in a conservative amino acid exchange at position 121 from aspartic acid to asparagine, which corresponds to an extracellular domain of MPZ responsible for its adhesive properties. We implicate this mutation as underlying the CMT phenotypes observed in this family for the following reasons: (1) All affected members contained the mutation, whereas unaffected members did not. (2) The mutation was not observed in 200 healthy controls without neuropathy. (3) Provean, Mutation Taster, and PolyPhen2 all predicted that the mutation was likely damaging. (4) The amino acid is highly conserved in vertebrates; is juxtaposed with known mutations (reported as Tyr119Cys [Senderek et al., 2000]), such as Asn122Ser; and is located in the critical disulfide bonding domain (between Cys-50 and Cys-127) responsible for the adhesive properties of the protein. (5) Lastly, the mutation occurs in an extracellular MPZ domain, producing a polarity variation that may consequently affect the hydrogen bonds responsible for the correct folding and dimerization of the protein (**Supplemental Figure 2**).

## Conclusion

The identification of a novel mutation in MPZ in this family expands our knowledge of the genetics of CMT2. The Asp121Asn mutation identified in this study together with the detailed clinical description of the affected family members broadens the phenotypic spectrum of mutations in the extracellular domain of MPZ and emphasizes the influence of other modulatory factors in the expression of disease pathology.

## Author Contributions

DF and JJ conceived this study and provided financial support. DF, JJ, and XD designed the study. WG, YH, RW, HW, and SS participated in study design and sample collection. XD and WG managed data. DF and JJ were responsible for project management. XD, JJ, and DF prepared and revised the manuscript. XD, JJ, and DF had key roles in the study.

## Conflict of Interest Statement

The authors declare that the research was conducted in the absence of any commercial or financial relationships that could be construed as a potential conflict of interest.

## Footnotes

1 http://www.hgmd.org/

2 http://www.ncbi.nlm.nih.gov

3 http://www.ncbi.nlm.nih.gov/projects/SNP

4 http://www.molgen.ua.ac.be/CMTMutations/

5 http://www.hgvs.org

6 http://provean.jcvi.org/index.php

7 http://genetics.bwh.harvard.edu/pph2/index.shtml

8 http://www.mutationtaster.org/

## Supplementary material

SUPPLEMENTAL FIGURE 1 **Axial and coronal T1-weighted images of the lower limbs. Proband III-5 (C, F) exhibited fatty infiltration, including massive fatty atrophy of the anterolateral compartments and gastrocnemius muscles.** His son, IV-7 **(B, E)** displayed subtle and symmetric fatty infiltration of the soleus and gastrocnemius muscles. **(A)** and **(D)** depict the normal appearance of the lower leg musculature of a healthy control.

SUPPLEMENTAL FIGURE 2 **The Asp121Asn mutation is predicted to change the protein structure by introducing a neutral-polar amino acid from a polar charge.** Asp121 has acidic features and acts as a hydrogen bond acceptor because of its side chain carboxylic function. Conversely, Asn121 is basic and may act both as a hydrogen bond acceptor and donor based on its side-chain amidic nitrogen. Thus, Asp121Asn may affect the hydrogen bonds responsible for the folding and dimerization of the α-helix structure. However, Asp121Asn does not dramatically change the tertiary protein structure, which results in late-onset instead of early onset axonal neuropathy.
