# Supplementary figures and images for: A Novel Asp121Asn Mutation of Myelin Protein Zero Is Associated with Late-Onset Axonal Charcot-Marie-Tooth Disease, Hearing Loss and Pupil Abnormalities
Source: Front Aging Neurosci. 2016 Sep 22;8:222. doi: 10.3389/fnagi.2016.00222 (PMC5054897; doi:10.3389/fnagi.2016.00222)

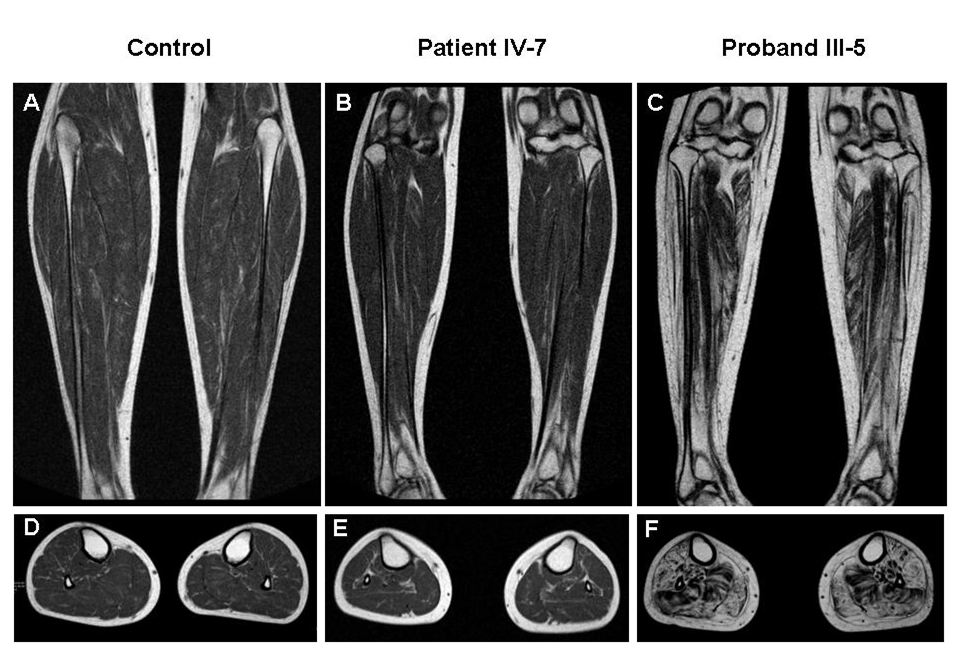

Supplement: SUPPLEMENTAL FIGURE 1 — Axial and coronal T1-weighted images of the lower limbs. Proband III-5 (C, F) exhibited fatty infiltration, including massive fatty atrophy of the anterolateral compartments and gastrocnemius muscles. His son, IV-7 (B, E) displayed subtle and symmetric fatty infiltration of the soleus and gastrocnemius muscles. (A) and (D) depict the normal appearance of the lower leg musculature of a healthy control. [file Image_1.TIF]

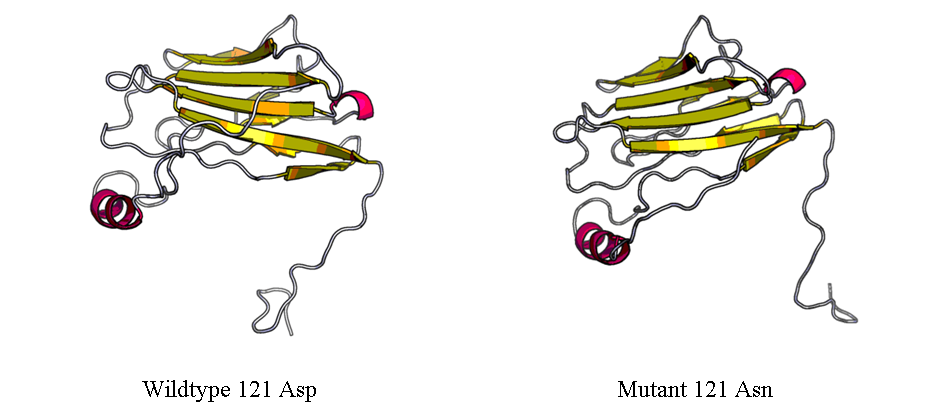

Supplement: SUPPLEMENTAL FIGURE 2 — The Asp121Asn mutation is predicted to change the protein structure by introducing a neutral-polar amino acid from a polar charge. Asp121 has acidic features and acts as a hydrogen bond acceptor because of its side chain carboxylic function. Conversely, Asn121 is basic and may act both as a hydrogen bond acceptor and donor based on its side-chain amidic nitrogen. Thus, Asp121Asn may affect the hydrogen bonds responsible for the folding and dimerization of the α-helix structure. However, Asp121Asn does not dramatically change the tertiary protein structure, which results in late-onset instead of early onset axonal neuropathy. [file Image_2.TIF]
